# Gray and White Matter Contributions to Cognitive Frontostriatal Deficits in Non-Demented Parkinson's Disease

**DOI:** 10.1371/journal.pone.0147332

**Published:** 2016-01-19

**Authors:** Catherine C. Price, Jared Tanner, Peter T. Nguyen, Nadine A. Schwab, Sandra Mitchell, Elizabeth Slonena, Babette Brumback, Michael S. Okun, Thomas H. Mareci, Dawn Bowers

**Affiliations:** 1 Department of Clinical and Health Psychology, University of Florida, Gainesville, Florida, United States of America; 2 Department of Biostatistics, University of Florida, Gainesville, Florida, United States of America; 3 Department of Neurology, University of Florida, Gainesville, Florida, United States of America; 4 University of Florida Center for Movement Disorders and Neurorestoration, Gainesville, Florida, United States of America; 5 Biochemistry and Molecular Biology, University of Florida, Gainesville, Florida, United States of America; Nathan Kline Institute and New York University School of Medicine, UNITED STATES

## Abstract

**Objective:**

This prospective investigation examined: 1) processing speed and working memory relative to other cognitive domains in non-demented medically managed idiopathic Parkinson’s disease, and 2) the predictive role of cortical/subcortical gray thickness/volume and white matter fractional anisotropy on processing speed and working memory.

**Methods:**

Participants completed a neuropsychological protocol, Unified Parkinson’s Disease Rating Scale, brain MRI, and fasting blood draw to rule out vascular contributors. Within group *a priori* anatomical contributors included bilateral frontal thickness, caudate nuclei volume, and prefrontal white matter fractional anisotropy.

**Results:**

Idiopathic Parkinson’s disease (n = 40; Hoehn & Yahr stages 1–3) and non-Parkinson’s disease ‘control’ peers (n = 40) matched on demographics, general cognition, comorbidity, and imaging/blood vascular metrics. Cognitively, individuals with Parkinson’s disease were significantly more impaired than controls on tests of processing speed, secondary deficits on working memory, with subtle impairments in memory, abstract reasoning, and visuoperceptual/spatial abilities. Anatomically, Parkinson’s disease individuals were not statistically different in cortical gray thickness or subcortical gray volumes with the exception of the putamen. Tract Based Spatial Statistics showed reduced prefrontal fractional anisotropy for Parkinson’s disease relative to controls. Within Parkinson’s disease, prefrontal fractional anisotropy and caudate nucleus volume partially explained processing speed. For controls, only prefrontal white matter was a significant contributor to processing speed. There were no significant anatomical predictors of working memory for either group.

**Conclusions:**

Caudate nuclei volume and prefrontal fractional anisotropy, not frontal gray matter thickness, showed unique and combined significance for processing speed in Parkinson’s disease. Findings underscore the relevance for examining gray-white matter interactions and also highlight clinical processing speed metrics as potential indicators of early cognitive impairment in PD.

## Introduction

The cognitive features of Parkinson’s disease (PD) are commonly attributed to frontostriatal dysfunction [1–4]. Frontostratial dysfunction can manifest as 1) slowness of thinking or information processing that reduces one’s ability to complete time based tasks efficiently; 2) mental inflexibility/working memory difficulty; 3) verbal or motor response suppression/disinhibition; and 4) abstract reasoning or problem solving difficulties. PD cognitive difficulties can, however, involve other cognitive realms such as the ability to learn new information (declarative memory; encoding/ retrieval; e.g., [5, 6]); language related operations, often measured with tests of verbal fluency and sentence comprehension [7]; and a wide variety of visuospatial skills [1]. Some researchers [1, 6, 7], yet not all [8], suggest these additional PD cognitive deficits (i.e., declarative memory, language, visuospatial) are subordinate to frontostriatal deficits. Further investigation into cognitive frontostriatal functions in PD and their relative contribution to the other cognitive domains will help clarify this issue.

Neuroanatomically, cognitive frontostriatal functions are linked to the frontostriatal circuitry that involves cortical gray matter, white matter, and subcortical gray matter regions [1]. Frontostriatal circuits originate in the frontal cortex, project to the striatum (caudate/ putamen; components of the basal ganglia), and then return to their respective cortical origin via the thalamus [9–11]. There are at least five circuits separated into ‘motor’ and ‘complex’ function, with the frontostriatal cognitive circuit involving projections from the dorsolateral prefrontal cortex to the dorsal portion of the caudate [11–15]. Dopaminergic, serotonergic, noradrenergic, and cholinergic cell groups along these pathways facilitate communication between the frontostriatal gray-white matter components [16–18].

Cognitive frontostriatal functions associate with separate regions of the frontostriatal circuitry. For example, among non-demented healthy adults, information processing speed abilities have largely been linked to prefrontal white matter integrity as measured by diffusion imaging [19, 20]. By contrast, animal/human lesion studies reveal working memory’s primary cortical site in the prefrontal cortex [21–23]. Fluid abstract reasoning, problem solving, and error monitoring involves various regions of the frontal cortex [24–26], although exceptions are noted [27].

For non-demented individuals with PD, the type and severity of frontostriatal cognitive deficit (e.g., processing speed, working memory, inhibition, abstract reasoning), relative to the integrity of other cognitive domains (e.g., language, visuospatial, memory) should speak to the location and burden of the disease. The six pathological stages of PD [28, 29] show that Lewy neurites and bodies progress from brainstem to neocortex. By stage three, there is disruption to the basal ganglia and associated dopaminergic pathways. Some investigators have shown that processing speed associates with caudate nucleus integrity [30–33]. Other investigators exclusively examining white matter integrity in PD report associations between white matter and processing speed, working memory, inhibition, and/or problem solving [34–38]. Abstract reasoning impairment as well as impairments in other cognitive domains (e.g., visuospatial) may signify cortical gray matter loss [39–44]. With minimal exception [37], however, these studies are limited in that they have examined PD cognition with either gray or white matter analyses. We propose that examining the relative contribution of gray and white matter integrity to the cognitive frontostriatal profile in PD will improve understanding of cognitive-neuroanatomical correspondence associated with disease progression.

The current study prospectively recruited individuals with idiopathic PD as well as non-PD “control” peers to assess hypotheses regarding 1) the dominance of frontal-striatal deficits in non-dementia PD and 2) the relative importance of cortical gray matter, white matter, and subcortical gray matter on these deficits. Using clinical neuropsychological tools, we expected individuals with PD to show dominant impairment in processing speed and working memory relative to non-PD peers, and for processing speed and working memory abilities to explain a portion of variability to other cognitive domains such as learning/memory, visuospatial, and motor abilities. Based on the rationale that frontostriatal deficits in PD are largely based on caudate nucleus to frontal cortical gray matter connections, we examined the relative contribution of cortical gray, white matter, and subcortical gray regions on the processing speed and working memory scores of both groups. Potential covariates that could confound white matter or frontostriatal functions (e.g., vascular risk factors) were also examined.

## Materials and Methods

### Participants

All participants had to be right-handed [45], show no signs of dementia (telephone screening for cognitive impairment had to be > 34; [46]; in-person completion of the Dementia Rating Scale-Revised (DRS-2; [47]) score > 130), and speak fluent English. Individuals with PD were diagnosed by a movement disorder trained neurologist, met criteria outlined by the UK Parkinson’s Disease Society Brain Bank Clinical Diagnostic Criteria [48] and had a Hoehn and Yahr scale [49] ranging from 1–3. All participants were tested while on-medication. Medical exclusions included having any underlying disease likely to limit lifespan or outcome analysis: cancer requirement treatment in the past 5 months (exception; non-melanoma skin cancer), serious infectious diseases (e.g., self-reported HIV), myocardial infarction or cerebrovascular accident, congestive heart failure, chronic hepatitis, history of organ transplantation, and any other condition likely to limit lifespan, seizure disorders, head trauma resulting in intensive care. Surgical exclusions included having undergone Deep Brain Stimulation. Neurodegenerative exclusions included evidence of secondary/atypical Parkinsonism as suggested by the presence of any of the following: 1) history of major stroke(s) associated with cognitive sequelae, 2) exposure to toxins or neuroleptics, 3) history of encephalitis, 4) neurological signs of upper motor neuron disease, cerebellar involvement, supranuclear palsy, or significant orthostatic hypertension. Psychiatric exclusions included a major psychiatric disorder as assessed by the psychiatric or neurological team. We did not exclude for depression or anxiety because many PD patients report such symptoms. We excluded individuals with conditions likely to affect cognitive or MRI testing such as claustrophobia, non-medical bodily metal, pace-maker device, less than five years of education, inability to read or write, self-reported hearing difficulty that interferes with standardized test administration.

### Procedures

The study was approved by the University of Florida Health Center Institutional Review Board (Protocol #472–2007). Written consent was obtained from all participants and the research followed the Declaration of Helsinki. Recruitment was conducted with a yoked procedure such that individuals were recruited first and non-PD “controls” peers second. Recruitment for individuals with PD involved a combination of: 1) brochure mailings to individuals identified through a clinical-research database within our local movement disorder center; 2) direct physician referrals, 3) advertisement at different PD support symposiums. Control participants were recruited through mail-outs to targeted individuals in two counties who met demographic inclusion criteria, community fliers, and free community memory screenings. All individuals were screened via telephone or in person, and then completed a baseline cognitive testing to ensure cognitive criteria. While on-medication participants completed neuropsychological testing, MRI, fasting blood draw, and the Unified Parkinson’s Disease Rating Scale (UPDRS; [50]). A spouse/friend completed participants’ instrumental activity of daily living questionnaire [51]. Measures of comorbidity [52], depression, apathy [53], and anxiety [54], were examined as potential covariates. Medications were reverted to a common metric (Levodopa Equivalency Dose; LED [55]). Raters blind to diagnosis double scored and entered all data.

### Neuropsychological Test Measures by Domain

Raw test scores were standardized to z-scores based on demographically similar published norms [56–58]. Individual test z-scores were then averaged into z-score composites. The composite groupings were based on cognitive domains discussed within clinical neuropsychological literature [59]. *General cognitive abilities*–DRS-2 (total score [47]). *Premorbid Intellectual Estimate* -Wechsler Test of Adult Reading (WTAR; total score correct [60]). *Attention*—Wechsler Memory Scale-Third Edition WMS-III Digit Span forward (longest span forward) and Spatial Span forward (total score) subtests [61]; *Processing speed*–Trail Making Test Part A (time to completion [62]), WAIS-III Digit Symbol (score within 120 seconds [63]); Stroop Color Word Test—Word Reading test condition (score within 45seconds [64]); *Working Memory*–Wechsler Memory Scale-III [61] Digit Span Backward Span (longest span backward), Spatial Span Backward (total score), and Letter Number Sequencing (total score); *Inhibition/set shifting-* Trail Making Test Part B minus Part A (based on standardized scores created from time to completion [65–67]), Stroop Color Word Test—Color-Word Condition (total correct [64, 68]). *Higher Abstract Reasoning/Problem Solving*—WASI-III Matrix Reasoning subtest [58], Tower Test (total achievement score [69]); Wisconsin Card Sorting Test (total errors [70]); *Language/ Lexical Retrieval*—Boston Naming Test (total correct [71]); Animal Fluency (total correct); Letter Fluency (total correct [72, 73]); *Visual Perceptual and Spatial*—Benton Face Recognition (total correct [74, 75]); Judgment of Line Orientation (total correct [76, 77]); *Learning and Memory*—WMS-III Logical Memory [61] subtest (delay free recall) and Visual Reproduction subtest (delay free recall & recognition); *Motor speed*—Finger Tapping Test (dominant, non-dominant taps [57, 78]).

### MRI Protocol

The MRI protocol was designed to provide information regarding subcortical volume, cortical volume, and white matter integrity. The brain MRI was conducted within 24 hours of cognitive testing via Siemens 3T Verio and 8-channel head coil for: 1) Two T1-weighted scans (176 contiguous slices, 1mm^3^ voxels, TR/TE = 2500/3.77ms) optimized for gray/white matter segmentation; 2) Diffusion two separate single-shot EPI, gradients applied along 6 directions (b = 100s/ mm^2^) and 64 directions (b = 1000s/ mm^2^), 73 contiguous axial slices, 2mm^3^ voxels, TR/ TE = 17300/81ms); 3) T2-weighted 176 contiguous slices, 1mm^3^ voxels, TR/ TE = 3200/ 409ms; 4) Fluid Attenuated Inversion Recovery (FLAIR; 176 contiguous slices, 1mm^3^ voxels, TR/TE = 6000/395ms). Between-group registration and intensity-based metrics were examined with TRACULA [79]. For preprocessing, both T1-weighted sequences per participant were entered into the FreeSurfer processing stream in order to correct for motion artifacts and improve segmentation quality. Both diffusion sequences per participant were merged together to create an acquisition with 70 non-overlapping directions (64 b = 1000 and 6 b = 100) and two b = 0 images. Effects of motion were partially corrected for using *eddy_correct* and then DTI metrics and registration with FreeSurfer processed T1 images were calculated for each participant.

#### Total Intracranial Volume (TICV in mm^3^)

The inner surface of the skull was acquired through FSL version 4.1—Brain Extraction Tool (BET [80]). An expert rater (Dice Similarity Coefficient; DSC > 0.99) manually cleaned the output on every sagittal slice. The inferior termination was based on a straight line between the bottom of the occipital bone and clivus.

#### Volumetric segmentation

FreeSurfer [81] was applied to acquire caudate nuclei, putamen, and thalamic volumes; trained raters blind to diagnosis corrected output for extraction errors. Final volumes were also segmented with FSL/FIRST [82] subcortical segmentation tool for comparison, with intraclass coefficient values between FreeSurfer and FIRST values ≥ 0.85.

#### Cortical thickness

FreeSurfer’s QDEC tool was applied *a priori* to acquire cortical thickness values to examine the relative contribution of regional cortical thickness to processing speed and working memory deficits. The QDEC group z map was full-width/half-max (FHWM) smoothed at 10mm. The difference map was projected onto an average brain for visualization. Corrections for multiple comparisons were performed using a Monte Carlo simulation with thresholding set at p = 0.01. FreeSurfer average cortical thickness for bilateral frontal, temporal, and parietal cortices were extracted for regression analyses.

#### Group Fractional Anisotropy (FA)

Tract Based Spatial Statistics [83] was applied with nonlinear registration aligning FA images to a group-specific (n = 80) FA template [80] affine aligned to a template space (MNI152). Files were merged for a mean FA skeleton (threshold of 0.2). Two group t-test comparisons (PD < Controls and PD > Controls) used a Threshold-Free Cluster Enhancement with 20,000 Monte Carlo permutations. Significance was set at p≤0.05, corrected for multiple comparisons.

#### FA within group metrics

The cortical parcellations from FreeSurfer (Desikan-Killiany Atlas) were combined to create lobar parcellations by hemisphere. Then the FreeSurfer tool *mri_aparc2aseg* was used to segment temporal lobe white matter up to 5 mm away from the gray-white boundary. Left and right temporal lobe white matter were extracted separately and checked for segmentation accuracy. A similar process was repeated for left and right parietal lobe. White matter masks were then rigid body transformed into diffusion space. Finally, *fslstats* was used to calculate mean FA within the ROIs. *For prefrontal white matter*: White matter was extracted with FreeSurfer, transformed to MNI152 space using a linear transformation (12 degrees of freedom), and cleaned in ITK-SNAP to acquire pre-frontal regions using the ventricular surface of the rostrum of the corpus callosum as a guide. The rater went posterior one slice coronally and removed four whole coronal slices of the white matter mask. Using the scalpel tool in ITK-SNAP, a trained rater removed the posterior portion of the brain and remaining temporal lobe. The masks including frontal white matter anterior to the rostrum of the corpus callosum were back transformed into FreeSurfer original space and then transformed into diffusion space using the registration matrix produced by the script *dt_recon*. *FSLstats* was used to extract mean FA within the regions of interest for each participant.

### Vascular Risk Markers

In order to characterize the relative contribution of potential vascular factors to the white matter and cognitive profile differences in the PD relative to the non-PD group we acquired the following: 1) *Fasting Blood Draw*–for cardiovascular and inflammatory markers (homocysteine, C-reactive protein; Uric Acid); 2) *Blood Pressure*–for systolic and diastolic metrics after a five-minute resting period; pulse pressure calculated from systolic BP minus diastolic BP; 3) *Leukoaraiosis* (*LA* [84])–white matter abnormalities were quantified by a reliable rater (DSC intra-rater range = 0.84–0.93; mean ± s.d. = 0.84 ± 0.12; Inter-rater range = 0.80–0.83) using an in-house macro within ImageJ [85, 86] on FLAIR scans; dependent variables = LA mm^3^ and LA relative to TICV.

### Statistical Analysis Approaches

SPSS version 22 was used for all analyses. Subcortical structures were corrected to TICV (e.g., Caudate/ TICV) and averaged across hemispheres. Independent t-tests examined group demographics. An analysis of variance assessed for a Group by Domain differences with follow-up analyses completed with independent t-tests. Independent t-tests examined group differences in neuroanatomical regions of interest. Mann-Whitney U tests compared C-reactive protein and UPDRS scores. Two-tailed Pearson correlations examined relationships between cognitive composites, neuroanatomical, and mood variables. Bonferroni corrections applied and commented upon when appropriate. Spearman rho correlations examined UPDRS-III and disease duration. Linear regression analysis was used to examine the contribution of *a priori* selected variables on age and education standardized composites of processing speed and working memory. Age was entered as a covariate in step1 of the model, with step 2 including the neuroanatomical cortical gray, white matter FA, and subcortical structures of interest. To explore associations between total caudate nucleus and prefrontal white matter integrity, separate partial correlations controlling for age examined caudate FA relative to white matter FA within each group. Fisher r-to-z transformation was conducted to examine statistical difference between coefficient values. Significance was set at 0.05. The wording ‘not significantly different’ is used when there is no statistical significance.

## Results

Of 186 people phone screened, 43 individuals with PD and 41 non-PD peers met criteria. Four enrolled participants could not complete MRI (i.e, claustrophobia, metal artifact). Our final sample involved 40 individuals with idiopathic PD and 40 non-PD “controls”.

### Participant Demographic, General Cognitive, and Motor Characteristics

Table 1. Groups were not significantly different in demographics, comorbidity, premorbid intellect and general cognition estimates. All were independent in instrumental activities of daily living (i.e., telephone, financial management) with all but one PD individual independently managing medications. PD was largely unilateral tremor dominant (70% H&Y ≤ 1.5). PD reported more symptoms of depression, apathy, and anxiety (p values <0.01).

**Table 1:** Parkinson’s disease (PD) and non-PD “control” peers demographic, motor, general cognition, and mood characteristics.

Measure	PD (n = 40)	Non-PD (n = 40)	t, u, x^2^	p-value
Demographics				
Age	67.80 ± 5.44, 60/79	68.18 ± 4.64, 62/79	-0.33	0.74
Education	16.28 ± 3.03, 10/20	16.75 ± 2.35, 12/20	-0.78	0.44
Sex (M:F)	32:8	33:7	0.08	0.78
Handedness	0.45 ± 3.66, 12/24	1.20 ± 3.07, 13/24	-0.99	0.32
Charlson Comorbidity	0.30 ±0.72, 0/4	0.28 ± .61, 0/2	0.12	0.91
Motor				
UPDRS-III	17.58 ± 10.74, 3/46	2.75 ± 3.36*; 0/15	83.50	<0.001
H&Y	1.64±0.76, 1/3	--	--	--
Disease Duration (yrs)	7.50 ± 5.15, 0/26	--	--	--
< 10 years duration	33 of 40; 83%	--	--	--
l-Dopa Equiv. Score	685.79 ± 371.49; 0/1450	1.00 ± 6.32*, 0/40	--	--
Side of Onset	25 R / 14 L / 1 axial	--	--	--
General Cognition				
WTAR Est. IQ	107.35 ± 7.68, 81/118	108.80 ± 8.76, 86/119	-0.79	0.43
DRS-2 Total	139.43 ± 3.13, 131/144	140.20± 2.49, 133/144	-1.23	0.22
Mood				
BDI-II raw	2.33 ± 2.99, 0/28	9.03 ± 6.93, 0/16	-5.61	<0.001
Apathy Scale	19.18 ± 4.22, 2/20	11.90 ± 6.6, 2/26	-2.20	0.03
State Anxiety	34.80 ± 11.00, 20/74	28.20 ± 6.46, 20/47	-3.27	<0.01
Trait Anxiety	33.33 ± 9.98, 20/54	30.30 ±7.29, 20/53	-1.54	0.13

MMSE = Mini-Mental State Examination; DRS-2 = Dementia Rating Scale– 2^nd^ Version; WTAR = Wechsler Test of Adult Reading; UPDRS Total = United Parkinson’s Disease Rating Scale Total score; l-Dopa Equiv. Score = Levodopa Equivalent Score = Total Daily levodopa dosage intake in milligrams.

*One control was on levodopa for restless leg syndrome; BDI-2 = Beck Depression Inventory-2.

### Vascular Risk Considerations

Table 2. Consistent with literature [87, 88], PD systolic blood pressure and pulse pressure metrics were significantly lower than non-PD peer controls (both p values <0.001), with homocysteine levels significantly higher in PD and trending with LED (PD: r(40) = 0.296, p = 0.067). There were no group differences in LA controlling for total intracranial volume.

**Table 2:** Parkinson’s disease (PD) and non-PD ‘control’ peers vascular risk variables.

Measure	PD (n = 40)	Non-PD (n = 40)	t, *u*, x^2^	p-value
Homocysteine	12.23±4.40, 2.7/22.2	10.65±1.69, 7.4/14.7	2.76	0.03
C Reactive Protein	1.42±1.47, 0.20/17.60	2.02±2.79, 0.20/8.1	-1.62	0.11
Uric Acid	5.31±1.06, 2.0/8.2	5.78±1.10, 2.9/7.6	-1.91	0.06
BP Systolic	128.16±10.46, 107/149	136.63±11.26, 112/157	-3.49	0.001
BP Diastolic	76.29±7.17, 62/93	78.30±6.40, 66/97.50	-1.32	0.19
Pulse Pressure	51.87±7.55, 37.50/64.67	58.33±9.26, 40/75.33	-3.42	0.001
Leukoaraiosis raw mm^3^	4950±6748, 497/29694	4032.15±4477.81, 0/19978	0.72	0.48
Leukoaraiosis/TICV %	0.29±0.40, 0/2	0.25±0.28, 0/1	0.57	0.57

Homocysteine—expected range 5–15 μmol/L, with 7 (17%) of PD participants having cut-offs above the recommended cut-point; C-reactive protein expected range = 0–3 mg/L; Uric Acid expected range 3–7 mg/dL. BP = blood pressure (average of measurements if multiple recordings acquired; results similar if based on the first BP recording; Systolic, p = 0.001; Diastolic, p = 0.14; Pulse Pressure, p = 0.001). Leukoaraiosis/TICV % = Percent of Leukoaraiosis as a proportion of total intracranial volume.

### PD and Non-PD Neuropsychological Comparisons

#### Group comparisons by cognitive domain

**Fig 1:**
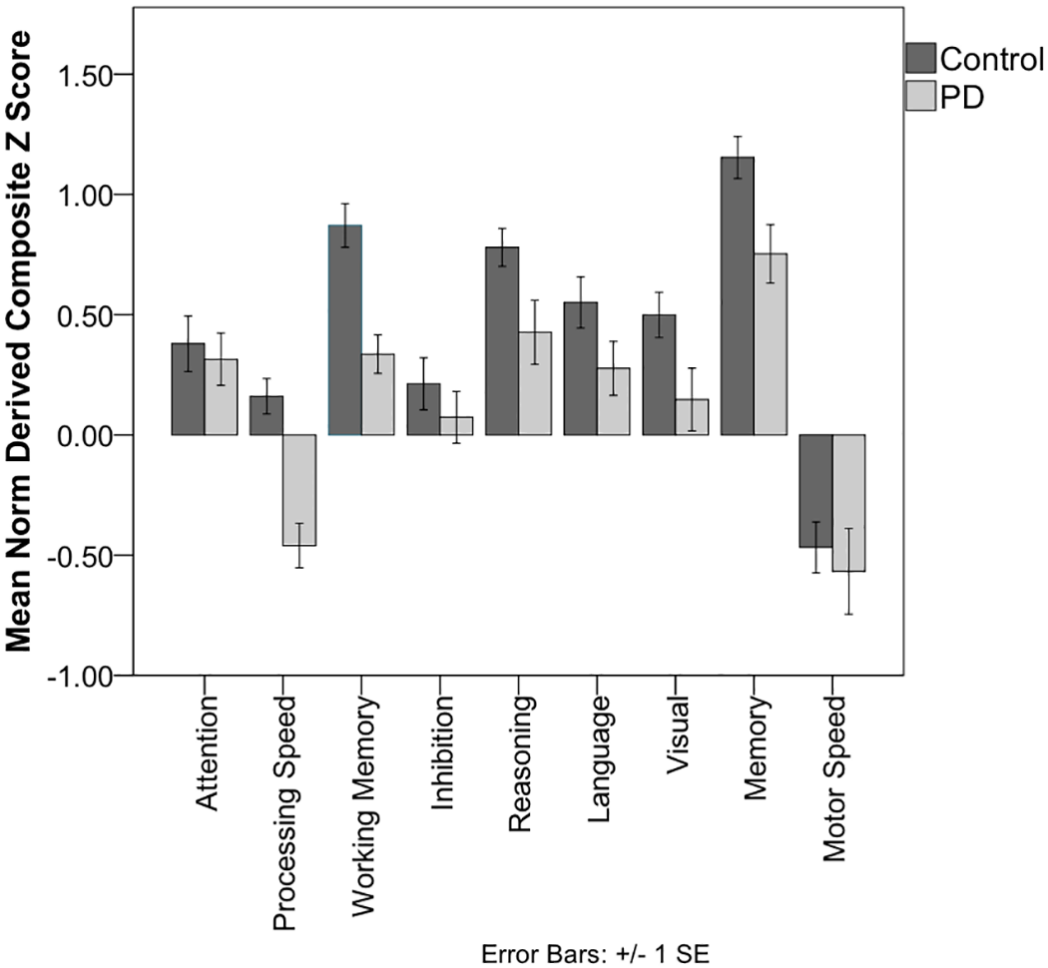
Group comparison by neuropsychological domain composite score. Each composite is based on the average of subtest standardized z-scores derived from published normative references. Average scores range from -0.67 to +0.67. Composite subtest scores are shown in S1 Table.

For all domains, mean score composites were within the average range relative to published norms for both the PD and non-PD (Table 3; Fig 1). A repeated measures analysis of variance identified a significant difference between group (F(1, 78) = 14.65, p<0.001) with a trend for a group by domain interaction (F(8, 624) = 1.89, p = .06). Follow-up between group comparisons showed significantly reduced processing speed and working memory composite scores in the PD relative to non-PD (p values < 0.001), with significant differences for composite subtests (p values <0.001; S1 Table). Individual PD impairment relative to normative standards occurred for the processing speed composite, however (borderline impaired (5.00%; 2/40)); Low average (35.00%; 14/40). For working memory, all PD participants scored at average levels or higher. Reasoning, visual perceptual/spatial, and memory composite scores were also significantly reduced in PD but with variability by subtest (e.g., significantly reduced delay memory (p values < 0.02) but not recognition; see S1 Table).

**Table 3:** Standardized neuropsychological composites for PD and non-PD peers with mean, standard deviation, and minimum/maximum scores shown.

	PD (n = 40)	Non-PD (n = 40)	t	p-value
Attention	0.31± 0.69, -1.39/2.12	0.38±0.73, -1.39/1.51	0.40	0.69
P. Speed	-0.48±0.61, -1.76/0.83	0.16±0.47, -0.58/1.27	5.25	<0.001*
W. Memory	0.34±0.51, -0.59/1.77	0.87±0.57, -0.45/1.92	4.42	<0.001*
Inhibition	0.07±0.68, -1.25/1.60	0.21/0.69, -1.10/1.45	1.12	0.27
Reasoning	0.43±0.84, -0.93/2.52	0.78/0.50, -0.36/1.76	2.28	0.03
Language	0.28±0.71, -1.17/1.73	0.55±0.67, -0.63/2.30	1.77	0.08
Visual	0.15±0.83, -2.50/1.34	0.50±0.59, -0.64/1.59	2.91	0.03
Memory	0.75±0.77, -0.67/2.33	1.15±0.55, 0.11/2.11	2.67	0.01
Motor	-0.57±1.12, -2.50/1.85	-0.47±0.67, -1.80/1.25	0.48	0.63

W. Memory = Working Memory; P. Speed = Processing Speed;

*Significant after Bonferroni correction. See S1 Table for the composite subtest raw and standardized scores by group.

Note: Further examination of the motor speed scores showed that group motor tapping scores were normally distributed (skewness; PD = 0.493; non-PD = 0.029), but different in range. For PD: although fifteen of the participants (38%) scored within the below average to impaired range, 10% (4 / 40) scored in the superior range. These superior scores associated with lower LED metrics (three had LED < 400; one of 700) and a disease range of 3 to 8 years. By contrast, for the non-PD peers 10% (4/40) scored in the below average with none in the superior range. Removing the four individuals with PD who had superior finger tapping scores showed a trend for finger tapping difference (t(73) = 1.78, p = .079; PD mean = -0.79).

#### Processing speed and working memory composite correlations to other cognitive domains

S2 Table. Processing Speed: PD processing speed positively associated with basic attention, language, visual perceptual/spatial, and finger tapping motor speed. Working Memory: PD working memory positively associated with inhibition, memory composites, attention and reasoning.

For non-PD, only working memory positively associated with the attention composite.

#### Processing speed and working memory composite correlations to motor and disease metrics

Processing speed associated with PD motor speed finger tapping scores (r(40) = 0.44, p<0.01), but not UPDRS part III (spearman rho (40) = -0.29, p = 0.069). Neither processing speed nor working memory significantly associated with years of disease duration, LED, mood metrics, or vascular variables (p values > 0.13).

### PD to Non-PD Neuroanatomical Comparisons

Between-group registration and intensity-based metrics demonstrated no significant group differences in diffusion sequence motion (Registration: average translation: t = 0.98, p = 0.33; average rotation: χ^2^ = 1.25, p = 0.26; Intensity: Percent bad slices χ^2^ = 0.26, p = 0.61; Average dropout score χ^2^ = 0.26, p = 0.61) suggesting data were appropriate for group comparisons.

**Fig 2:**
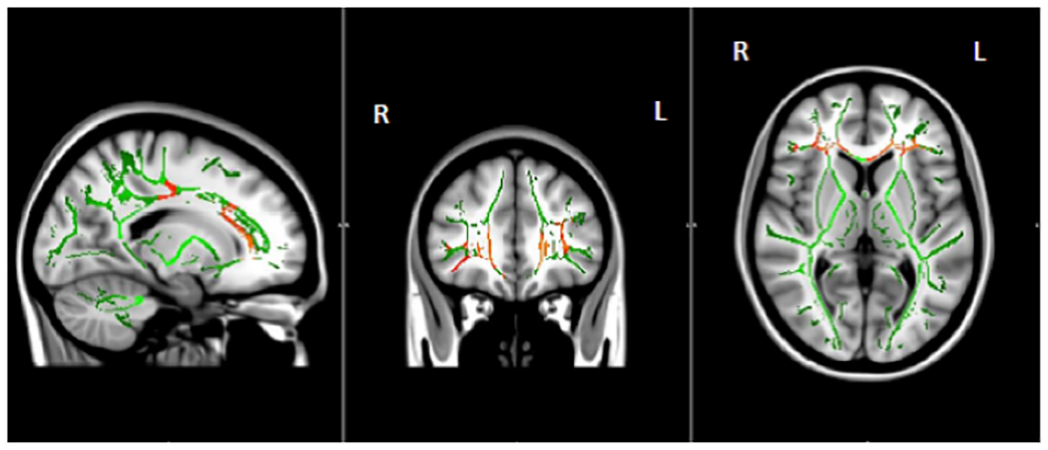
White matter areas with significantly decreased fractional anisotropy (FA) in PD (n = 40) versus non-PD peers (n = 40) corrected with threshold free cluster enhancement. Areas with significantly decreased FA are shown in colors ranging from red to yellow (p < 0.05, corrected for multiple comparisons). Voxelwise group comparisons of FA were carried out using TBSS (Tract-Based Spatial Statistics, part of FSL). TBSS projects all participants’ FA data onto a mean FA tract skeleton (shown in green), before applying voxelwise cross-subject statistics. MRI conducted within 24 hours of cognitive testing. R = Right; L = Left.

Table 4. Fig 2. *TICV*—was significantly greater in PD (p<0.001). *Cortical Gray Thickness Group Differences* (Fig 2)—were statistically similar for whole brain comparisons. Individual lobe mean thickness comparisons showed a trend (Bonferroni uncorrected) for reduced temporal thickness in PD (t(78) = -1.97, p = 0.053). *White Matter Fractional Anisotropy* (Fig 2; S1 Video)—as measured by voxel by voxel comparisons showed PD with reduced FA in specific tracts (corpus callosum genu and body, forceps minor, anterior thalamic radiations, portions of inferior fronto-occipital fasciculus, and uncinate fasciculus). Averages across lobe region (frontal, temporal, parietal) were, however, were not statistically different between groups (p values >0.13). *Subcortical Gray Volume*—comparisons were only significant for bilateral putamen (PD< Non-PD, p<0.001; remaining significant after Bonferroni correction) with this associating with disease duration (spearman rho (40) = -0.454, p = 0.003).

**Table 4:** Raw and TICV corrected neuroanatomical regions of interest for PD and non-PD peers with mean± standard deviation, and minimum/maximum scores shown). Significance noted by *.

	PD (n = 40)	Non-PD (n = 40)
Cortical		
Prefrontal	2.32±0.09, 2.11/2.56	2.31±0.08, 2.06/2.51
Thickness (mm)		
Temporal	2.64±0.09, 2.39/2.82	2.67±0.07, 2.49/2.81*
Parietal	2.24±0.09, 2.09/2.41	2.23±0.09, 2.03/2.38
White Matter		
Prefrontal	0.33±0.03, 0.25/0.38	0.33±0.02, 0.28/0.37
Mean FA		
Temporal	0.30±0.02, 0.24/0.34	0.30±0.02, 0.21/0.34
Parietal	0.34±0.02, 0.28/0.38	0.35±0.02, 0.30/0.38
Subcortical		
Caudate raw	7367±1164, 4754/10082	7086±992, 5452/10455
Raw mm^3^		
Putamen raw	9875±1148, 7958/13563	10221±1057, 8359/12570
Thalamus raw	13937±1313, 11294/18378	13120±1319, 10286/16222**
Subcortical		
% Caudate	0.43± 0.06, 0.30/0.55	0.44±0.05, 0.34/0.58
TICV corrected^a^		
% Putamen	0.507± 0.07, 0.44/0.72	0.64±0.06, 0.53/0.82**
% Thalamus	0.81±0.07, 0.64/0.97	0.82±0.08, 0.64/0.99
TICV cm^3^	1727±1698, 1416/2082	1603±1377, 1371/1881**

**p < .01;

*trend at p = .05;

^a^Bilateral subcortical structures corrected for total intracranial volume x 100. TICV = Total intracranial Volume in cm^3^. Using the non-PD mean and standard deviation TICV values, standardized z-scores indicated that 11/40 (27.5%) of the PD participants had a TICV at least one standard deviation above the control mean TICV, three (7.50%) individuals at least two standard deviations, and three (7.50%) at least three standard deviations larger than the control mean. For these reasons, subcortical volume structures are corrected for TICV. FSL/FIRST [82] subcortical values were not statistically different from FreeSurfer [81] with intraclass correlation > 0.85.

### Predicting Processing Speed and Working Memory in PD

#### Processing speed

Based on *a priori* hypotheses, we conducted regression analyses to explain the processing speed composite score in PD. The original model included total prefrontal gray thickness, prefrontal FA, and total caudate nucleus volume corrected for TICV as predictor variables. Due to potential associations with age on the anatomical structures, the variable of age in years was entered as a covariate in step 1 of each model.

**Fig 3:**
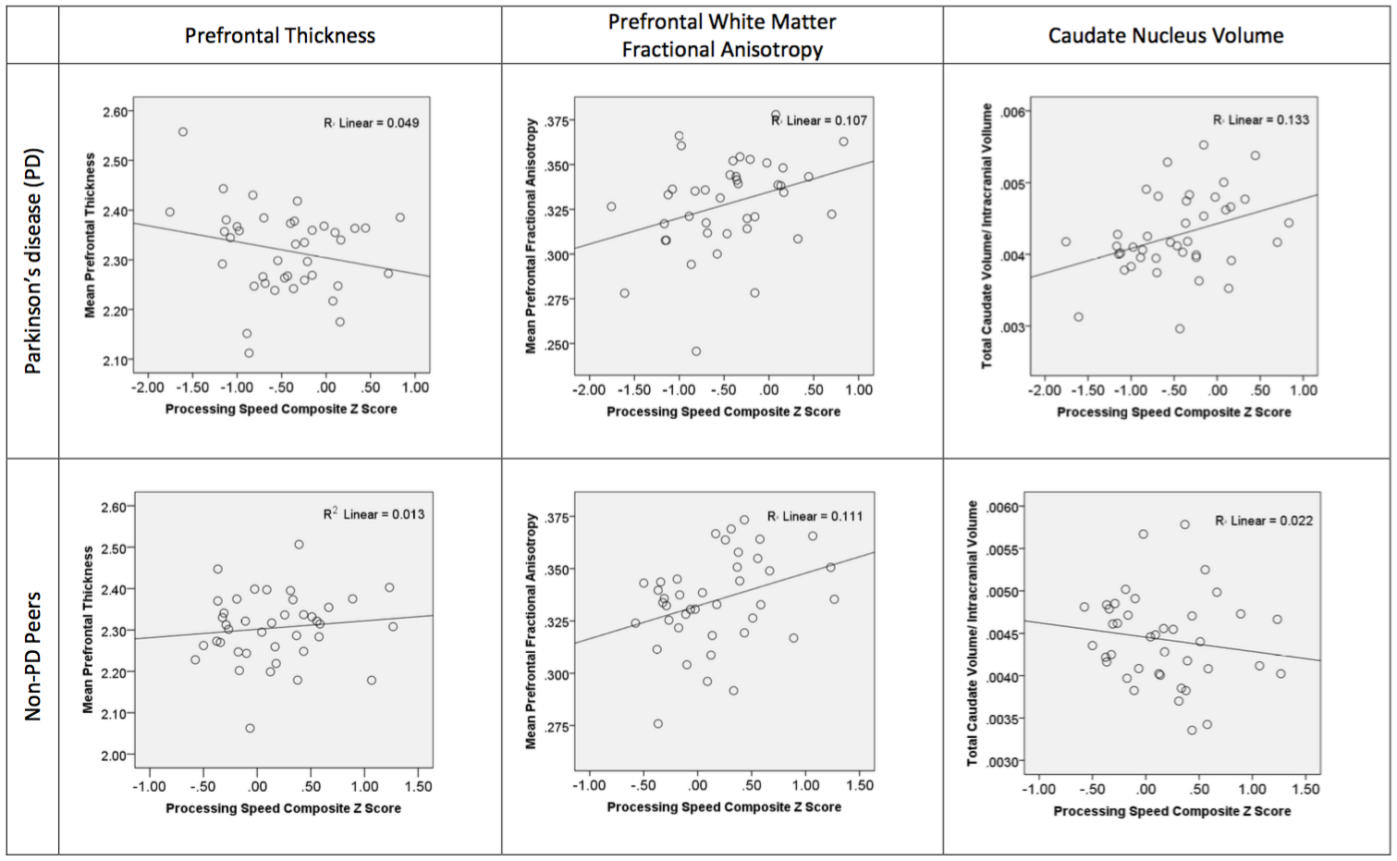
Scatter graphs showing group processing speed composite scores plotted against prefrontal gray thickness, prefrontal white matter fractional anisotropy, and caudate nucleus volume. The top row shows scatter graphs for the individuals with PD (n = 40). The second row presents the non-PD peers (n = 40). The processing speed composite is based on the average of subtest standardized z-scores derived from published normative references. Note: The referenced r value is r squared.

*For PD*: We controlled for age before examining the relative contribution of prefrontal cortical thickness, prefrontal white matter FA, and caudate nucleus volume on processing speed. Age was not a significant contributor (R = 0.006, p = 0.97) in step 1. The combined predictors for step 2 of the model was significant (R = 0.52, R^2^ = 0.27, F change = 4.27, p = 0.01) with positive significant beta weights only for caudate nucleus volume and prefrontal FA (beta weights: caudate nucleus volume = 0.32, p = 0.04; prefrontal FA = 0.32, p = 0.04; frontal thickness = -0.20, p = 0.16). We then explored the relative contribution of other structures. Excluding the caudate from step 2 weakened the overall model (R = 0.35, R^2^ = 0.12, p = 0.09). Replacing the caudate nucleus with total putamen volume (beta weight = 0.16, p = 0.31) or thalamus volume (beta weight = 0.10, p = 0.55) showed overall model reduction, supporting the caudate nucleus as the most relevant subcortical contributor. Replacing prefrontal FA with parietal FA (beta weight = -0.01, p = 0.96) weakened the model, although there was a trend for temporal FA contribution (beta weight = 0.33, p = 0.07). Replacing prefrontal gray thickness with temporal or parietal thickness did not change the model (beta weights < 0.12). Fig 3.

*For Non-PD*: Age was not a significant predictor in step 1 (R = 0.08, p = 0.61). In step 2, only prefrontal white matter FA had a moderate beta weight (beta weight = 0.36, p = 0.03), but the overall model was not significant (R = 0.42, R^2^ = 0.17, F change = 2.33, p = 0.09; prefrontal thickness beta weight = 0.20, p = 0.23; caudate nucleus beta weight = -0.21, p = 0.21).

Vascular risk blood variables and LA did not significantly contribute to the models for either group (all p values >0.11).

#### Working memory

For PD, age was not a significant contributor to the model (R = 0.20, p = 0.22). The anatomical variables of interest also not significantly predicting over that of age (F change = -0.61, p = 0.61; R = 0.30, R^2^ = 0.09, p = 0.51; beta weights Prefrontal Thickness = 0.14, Prefrontal FA = 0.04, Caudate = 0.18, all p values >0.31). Non-PD analyses were similar (Age R = 0.02, p = 0.91; F change = 0.15, p = 0.94).

Age, LA and vascular risk variables did not significantly contribute to the models for either PD or non-PD (all p values >0.11).

#### Considerations for neuroanatomical structures on motor speed

Given the association between PD processing speed and motor speed (finger tapping), we examined the contribution of prefrontal cortical thickness, prefrontal FA, and caudate nucleus volume on the finger tapping motor speed composite. These neuroanatomical variables were not significant contributors (PD: R = 0.29; R^2^ = 0.01, p = 0.35; beta weights Prefrontal Thickness = -0.15; prefrontal FA = -0.04; caudate = 0.24, p = 0.14).

## Discussion

Our prospective investigation identified processing speed as the dominant frontostriatal cognitive weakness in non-demented individuals diagnosed with PD, and that caudate nucleus volume and prefrontal white matter fractional anisotropy were significant contributors to their processing speed performance. For participants with PD, working memory was a secondary weakness relative to non-PD peers—yet no anatomical area of interest significantly contributed to their performance scores. The frontostriatal functions of processing speed and working memory abilities also explained a significant portion of variance (up to 24%) for scores of cognitive domains including learning/ memory, abstract reasoning, and perceptual/spatial matching. Our findings support the following assertions: 1) frontostriatal dysfunction and particularly processing speed is a primary contributor to other areas of cognition in PD [1, 6, 7]; 2) in order to understand PD cognition we need to examine both gray and white matter neuroimaging regions of interest [37].

For the processing speed metrics, nearly half (40%) of the PD sample scored in the below average or impaired range (less than the 9^th^ %ile) and no PD participant scored greater than average levels (i.e., greater than 75^th^ %ile). By contrast, each non-PD control participant scored at average levels or higher. Only PD processing speed scores positively associated with composites of basic attention, language (word retrieval and confrontation naming), perceptual/visuospatial abilities, and motor speed (finger tapping). These findings fit with expectations; the clinical processing speed measures we used in this investigation (Digit Symbol, Trail Making Test Part A, and Stroop Color Word Test—Word Reading) require sustained and selective attention, rapid integration of visual and verbal stimuli, and rapid hand/oral motor output [89, 90].

Processing speed has long been considered a fundamental component of the human cognitive architectural system [20, 91] and deficits on processing speed are considered sensitive markers to disease or brain damage [89, 90]. Working memory [92–95] and response inhibition [96–98] have additional dependence upon prefrontal and parietal gray matter. Abstract reasoning and decision-making are higher cortical functions. Although highly dependent upon prefrontal and the associative cortices [99, 100], abstract reasoning and decision making can be derailed by impaired processing speed, inhibitory functions, and working memory impairments [101, 102]. For PD, processing speed deficits in the confines of other relatively intact cognitive abilities, therefore, may mark a crucial time for cognitive intervention. Understanding white and gray matter contributions to processing speed has diagnostic and intervention relevance.

Neuroanatomically, fractional anisotropy of the prefrontal white matter explained a significant and similar portion of processing speed variance within both participant groups (such that higher prefrontal FA associated with better processing speed scores). The relationship between processing speed and white matter damage is well documented as a process of aging [19, 20]. While, reductions in processing speed has been observed with increasing amounts of white matter abnormalities which represents an ‘insult’ to white matter integrity (leukoaraioasis (LA) [84, 103, 104]), LA was not a contributor to processing speed within the current investigation. Prefrontal white matter appears therefore appears to be a foundational component of processing speed regardless of group disease status.

Tract based group comparisons, however, showed discrete anterior areas of prefrontal FA reduction in the PD relative to non-PD peers. Consistent with others’ reports (e.g., [35, 105, 106]), PD FA was reduced relative to their peers within regions of the bilateral forceps minor, anterior thalamic radiations, inferior fronto-occipital fasciculus, and uncinate fasciculus. These regions are intricately connected to the caudate nucleus—the only subcortical gray structure uniquely contributing to processing speed in PD.

Architectural studies of rhesus monkey show that the frontostriatal fibers have two major components [107]. Initially, the fibers course along with the long association fibers traveling within the frontal-occipital fasciculus lying just rostral to the lateral ventricle. Then, the set separates with one set traveling to the subcallosal fasciculus of Muratoff eventually ending within the caudate nucleus and putamen. The other set of striatal fibers enters the external capsule thereby targeting the ventral part of the caudate nucleus, the putamen and claustrum. Thus, the disease specific determinant of processing speed for PD may stem largely from the caudate nucleus and not reside within the prefrontal white matter alone. This interpretation would align with research supporting the caudate dopaminergic hypothesis of early cognitive impairment in PD [108–110].

Consistent with other reports [40, 43, 111], our study did not find group differences in cortical gray matter thickness and or associations between areas of cognition and prefrontal cortical thickness. Pereira and colleagues conducted a comprehensive examination of cognition and cortical thinning of individuals with PD relative to healthy adults who were enrolled in the Parkinson’s Progression Markers Initiative [112]. For cognitively well individuals with PD, there was limited reduced cortical thinning within the right inferior temporal gyrus, but no thinning in the frontal regions. Only the participants classified as PD-MCI via the Movement Disorder Society Task Force guidelines [113] had thinning in superior frontal, temporal, parietal, and occipital regions relative to their non-PD peers. Also consistent with our findings, Periera and colleagues’ assessment of processing speed (Symbol digit modalities test) did not associate with any cortical areas in either PD or non-PD peers. Segura and colleagues also show no associations between measures of (symbol digit modalities subtest, trail making test part A) and cortical thickness [43]. Cortical gray matter and subcortical gray matter thickness and volume differences appear minimal until individuals with PD have classified to have mild cognitive impairment [40, 43, 44].

Regarding other cognitive domains in our PD sample, we identified secondary difficulties in working memory. Despite scoring lower than their non-PD peers on working memory, *no* PD participant scored lower than average levels. No neuroanatomical region of interest explained PD working memory scores. This finding may be partially due to the sample’s limited working memory score range and otherwise average cognition relative to normative references and age matched peers. Between group analyses also showed PD weaknesses among sub-measures of learning/ memory, abstract reasoning, and spatial matching. Although PD group performance on learning/memory, abstract reasoning, perceptual/spatial matching was partially explained by frontostriatal deficits, we encourage further examination into individual profiles to rule out smaller PD heterogeneous subsamples (see [5]).

We recognize study limitations. First, we recognize the cognitive and neuroanatomical patterns may differ if participants were assessed off medication [114]. Second, two of our individuals had longer than 10 years of disease duration. Excluding these individuals from the analyses, however, did not change our cognitive or neuroanatomical findings. Third, PD individuals in our sample had larger TICV. Larger TICV in PD is not well known, although it has been reported [115] and referenced as a potential genetic association [116]. Head size may be a random effect of selection, but requires further consideration relative to interactions on white and gray matter metrics. Fifth, we recognize that our controls had slow motor speed (approximately a half standard deviation below published norms). Aside from acknowledging that some individuals within the non-PD control group had osteoarthritis of the hands, we are currently unable to explain this reduced finger tapping motor speed in our control group.

We encourage future investigations examining anatomical contributions to PD processing speed deficits. We conducted a post-hoc analysis examining gray-white matter contributors to processing speed in PD after additionally controlling for motor speed. This analysis showed that prefrontal white matter remained a significant contributor to processing speed, while the contribution of caudate nucleus volume reduced to a trend level (p = 0.10). We know, however, that caudate nucleus volume does not have a significant relationship to motor speed in our PD sample. For these reasons, we encourage additional statistical modeling to understand relationships between gray-white matter anatomical regions, processing speed, and motor speed in PD. Future investigators are also encouraged to examine frontal white matter with more sophisticated metrics, such as generalized anisotropy [117]. Fractional anisotropy, the white matter metric studied in the current investigation, may misrepresent white matter integrity due to the numerous kissing/ crossing fibers within the frontal forceps [118]. Finally, future studies are also encouraged to examine regions of caudate nucleus (ventral/dorsal; see [119]) on processing speed but also the relative integrity of specific white matter connections between these cortical to ventral/dorsal caudate regions on PD cognitive frontostriatal functions.

Despite study weaknesses, there were numerous study strengths including a prospective recruitment and assessment procedure for both PD and non-PD, an *a priori* structural neuroimaging methodology that incorporates white and gray matter regions of interest, and consideration for potential confounders such as vascular contributions. The neuropsychological protocol usedclinical measures that have normative references for age and/or education based peers. This has an advantage of providing a reference point of impairment for both the individuals with PD but also the non-PD peers. Finally, the findings demonstrate the probable value for coupling structural based white matter diffusion and gray matter region analyses with neuropsychological assessment for disease monitoring and prediction purposes.

Overall, this investigation demonstrated the dominance of frontostriatal deficits and particularly processing speed in non-demented individuals with idiopathic PD. It additionally showed unique and combined significance for caudate nucleus volume and prefrontal white matter FA on PD processing speed deficits. The findings improve awareness of gray and white matter interactions on the cognitive symptoms PD. It also highlights the value of clinical processing speed metrics as potential indicators of early cognitive impairment in PD.

## Supporting Information

S1 Table Raw and normative based standardized neuropsychological scores for PD (n = 40 unless otherwise noted) and non-PD peers (n = 40 unless otherwise noted) with mean, standard deviation, and minimum/maximum scores shown. *PD<Non-PD peers p<0.05; **PD<non-PD peers p<0.01. DS = Digit Span Backward span length; SS = Spatial Span Backward total score; TMT = Trail Making Test; Tower Ach. = Tower Achievement score; WCST = Wisconsin Card Sorting Test; BNT = Boston Naming Test; FAS = Controlled Oral Word Association Test letters F, A, S; JLO = Judgment of Line Orientation; LM = Logical Memory; VR = Visual Reproductions; FT Dom = Finger Tapping Dominant hand; FT Nondom = Finger Tapping Non-dominant hand. ^a^PD n = 39 due to missing data; ^b^PD n = 39 and Control n = 38 due to color-blindness. All raw scores are unadjusted for age, sex, and education.
(DOCX)

S2 Table Separate group correlation matrices for processing speed and working memory relative to other composites. *p<0.05, **p<0.01; two-tailed.
(DOCX)

S1 Video 3D representation of FA differences between PD and non-PD peers. Yellow depicts regions of reduced fractional anisotropy in PD. White depicts mean FA skeleton.
(MP4)
