# Supplementary material for: Gray and White Matter Contributions to Cognitive Frontostriatal Deficits in Non-Demented Parkinson's Disease
Source: PLoS One. 2016 Jan 19;11(1):e0147332. doi: 10.1371/journal.pone.0147332 (PMC4718544; doi:10.1371/journal.pone.0147332)
Supplement: S1 Table — *PD<Non-PD peers p<0.05; **PD<non-PD peers p<0.01. DS = Digit Span Backward span length; SS = Spatial Span Backward total score; TMT = Trail Making Test; Tower Ach. = Tower Achievement score; WCST = Wisconsin Card Sorting Test; BNT = Boston Naming Test; FAS = Controlled Oral Word Association Test letters F, A, S; JLO = Judgment of Line Orientation; LM = Logical Memory; VR = Visual Reproductions; FT Dom = Finger Tapping Dominant hand; FT Nondom = Finger Tapping Non-dominant hand. aPD n = 39 due to missing data; bPD n = 39 and Control n = 38 due to color-blindness. All raw scores are unadjusted for age, sex, and education. (DOCX) [file pone.0147332.s001.docx]

**S1 Table. Raw and normative based standardized neuropsychological scores for PD (n=40 unless otherwise noted) and non-PD peers (n=40 unless otherwise noted) with mean, standard deviation, and minimum/maximum scores shown.**

|  |  | PD |  |  | Non-PD | |
| --- | --- | --- | --- | --- | --- | --- |
|  | Measure | Raw | Z-score |  | Raw | Z-score |
| Attention | DS Forward | 6.73±1.15, 4/9 | 0.35±0.83, -1.55/1.90 |  | 7.05±1.18, 5/9 | 0.57±0.82, -0.95/1.90 |
|  | SS Forward | 7.85±1.78, 4/12 | 0.28±1.05, -2.00/2.33 |  | 7.63±1.84, 2/11 | 0.18±1.06, -2.67/2.00 |
| Processing Speed | Digit Symbol | 50.08±13.22, 27/79 | -0.30±0.78, -1.67/1.33** |  | 60.70±11.24, 40/87 | 0.44±0.73, -1.00/2.00** |
|  | TMT A^a^ | 39.61±11.19, 20.80/73.60 | -0.46±0.59, -1.76/0.83** |  | 30.44±7.01, 17/46 | 0.15±0.71, -1.30/1.90** |
|  | Stroop Word^b^ | 85.51±15.11, 60/116 | -0.54±0.78,-2.00/1.10* |  | 93.66±12.38, 59/115 | -0.13±0.63, -2.10/0.70* |
| Working Memory | DS Backward | 4.73±1.15, 2/7 | 0.20±0.84, -1.65/2.30** |  | 5.60±1.19, 4/8 | 0.83±0.86, -0.35/2.30** |
|  | SS Backward^a^ | 6.79±1.40, 4/9 | 0.35±0.81, -1.33/2.00** |  | 7.70±1.52, 4/11 | 0.86±0.89, -1.33/2.67** |
|  | Letter-Number | 10.03±1.91, 6/14 | 0.47±0.70, -1.33/2.00** |  | 11.33±1.95, 7/16 | 0.92±0.61, -0.33/2.33** |
| Inhibition | Trail B-A time^a^ | 61.43±34.39,14/157 | 0.15±0.87, -1.30/1.70 |  | 39.70±20.80,9.80/98.10 | 0.28±0.95, -1.60/2.00 |
|  | Stroop CW^b^ | 33.31±9.73, 13/50 | 0.02±0.97, -1.70/1.90 |  | 34.47±7.40, 22/53 | 0.16±0.79, -1.80/2.30 |
| Reasoning | Matrix Reasoning | 21.90±6.40, 9/30 | 0.76±0.99, -1.10/2.20* |  | 24.63±4.77, 11/30 | 1.17±0.69, -1.00/2.10* |
|  | Tower Ach. | 17.63±3.40, 11/26 | 0.58±0.79, -0.67/2.67 |  | 19.18±3.39, 10/26 | 0.91±0.82, -1.00/2.67 |
|  | WCST errors^a^ | 36.26±24.77, 7/85 | -0.08±1.32, -2.30/3.00 |  | 25.90±17.67, 7/71.00 | 0.26±0.82, -1.40/1.70 |
| Language | BNT | 56.80±3.34, 44/60 | 0.65±0.97, -1.20/2.30 |  | 57.55±2.21, 52/60 | 0.77±1.00, -1.30/2.60 |
|  | Animal Fluency | 20.83±5.12, 11/31 | 0.25±1.00, -1.90/2.40 |  | 22.63±4.94, 15/37 | 0.62±0.99, -1.00/3.30 |
|  | FAS | 41.20±11.87, 18/65 | -0.07±1.00, -1.70/1.90 |  | 45.25±10.71, 25/70 | 0.26±0.93, -1.90/2.20 |
| Visual | Benton Faces | 42.78±4.97, 28/50 | -0.01±1.24, -3.00/2.10 |  | 44.58±3.64, 37/52 | 0.39±0.93, -1.50/2.10 |
|  | JLO | 25.25±4.30, 6/30 | 0.31±0.74, -2.00/1.08* |  | 26.93±2.48, 22/30 | 0.59±0.49, -0.74/1.08 |
| Memory | LM Delay | 24.63±7.98, 11/41 | 0.78±.99, -1.33/2.67* |  | 28.53±5.67, 18/39 | 1.24±0.66, 0.00/2.33 |
|  | VR Delay | 51.68±21.06, 13/88 | 0.74±1.07, -1.33/2.67* |  | 62.43±19.37, 26/99 | 1.29±0.93, -0.33/3.00 |
|  | VR Recog. | 42.73±3.31, 34/48 | 0.74±0.84, -1.00/2.33 |  | 43.33±3.21, 33/48 | 0.93±0.72, -1.00/2.00 |
| Finger Tapping | FT Dom | 46.08±7.90, 21.4/63.2 | -0.42±1.09, -2.60/2.70 |  | 47.04±5.76, 33.30/58.80 | -0.43±0.79, -2.30/1.30 |
|  | FT Nondom. | 39.72±8.64, 20.9/59.2 | -0.72±1.45, -3.00/3.60 |  | 41.76±5.26, 30.44/53.80 | -0.51±0.72, -2.00/1.40 |

*PD<Non-PD peers p<0.05; **PD<non-PD peers p<0.01

DS=Digit Span Backward span length; SS=Spatial Span Backward total score; TMT=Trail Making Test; Tower Ach.=Tower Achievement score; WCST=Wisconsin Card Sorting Test; BNT=Boston Naming Test; FAS=Controlled Oral Word Association Test letters F,A,S; JLO=Judgment of Line Orientation; LM=Logical Memory; VR=Visual Reproductions; FT Dom=Finger Tapping Dominant hand; FT Nondom=Finger Tapping Non-dominant hand

^a^PD n=39 due to missing data; ^b^PD n=39 and Control n=38 due to color-blindness. All raw scores are unadjusted for age, sex, and education
