# Supplementary material for: Gray and White Matter Contributions to Cognitive Frontostriatal Deficits in Non-Demented Parkinson's Disease
Source: PLoS One. 2016 Jan 19;11(1):e0147332. doi: 10.1371/journal.pone.0147332 (PMC4718544; doi:10.1371/journal.pone.0147332)
Supplement: S2 Table — *p<0.05, **p<0.01; two-tailed. (DOCX) [file pone.0147332.s002.docx]

**S2 Table. Separate group correlation matrices for processing speed and working memory relative to other composites**

| PD (n=40) | | | | | | | | | |
| --- | --- | --- | --- | --- | --- | --- | --- | --- | --- |
| Index | Attention | Processing Speed | Working Memory | Inhibition | Reasoning | Language | Visual | Memory | Motor speed |
| Processing Speed | **0.36*** | 1.00 | 0.21 | 0.21 | 0.20 | **0.38*** | **0.40*** | 0.19 | **0.44**** |
| Working Memory | **0.38*** | 0.21 | 1.00 | **0.50**** | **0.40**** | 0.12 | **0.32*** | **0.49**** | 0.10 |
| Non-PD (n=40) | | | | | | | | | |
| Index | Attention | Processing Speed | Working Memory | Inhibition | Reasoning | Language | Visual | Memory | Motor Speed |
| Processing Speed | 0.14 | 1.00 | 0.13 | 0.04 | -0.03 | -0.04 | 0.15 | 0.13 | 0.04 |
| Working Memory | **0.50**** | 0.13 | 1.00 | 0.23 | 0.20 | 0.14 | 0.23 | 0.19 | 0.11 |

*p<0.05, **p<0.01; two-tailed
